# Evaluation of biochar-based phosphate fertilizer for improving soil properties, chili yield, and microbial function

**DOI:** 10.3389/fpls.2026.1778669

**Published:** 2026-05-14

**Authors:** Xiaoqing Zhu, Fei Liu, Yingyuan Cen, Panfeng Tu, Lansheng Deng, Junjie Liang, Lifang Deng, Yong Chen

**Affiliations:** 1 Key Laboratory of Energy Plants Resource and Utilization, Ministry of Agriculture and Rural Affairs, Guangdong Engineering Technology Research Center for Agricultural and Forestry Biomass, College of Future Biomass, South China Agricultural University, Guangzhou, China; 2 Guizhou Phosphate (Group) Co., Ltd., State Key Laboratory of Green and Efficient Development of Phosphorus Resources, Guiyang, China; 3 College of Horticulture and Landscape Architecture, Zhongkai University of Agriculture and Engineering, Guangzhou, China

**Keywords:** acidic red soil, biochar, chili, microbial diversity, phosphate fertilizer

## Abstract

**Introduction:**

Excessive use of chemical fertilizers leads to soil degradation during chili cultivation, particularly in acidic red soils with low phosphorus (P) availability, which limits yield and quality.

**Methods:**

In this study, a tobacco stem–derived biochar-based phosphate fertilizer (P-BCL800) was developed to address this problem. Pot experiments with six treatments (blank control; P-BCL800 alone; traditional phosphate fertilizer; and 33%, 66%, and 100% P-BCL800 replacement) were conducted.

**Results and discussion:**

The results indicated that all three P-BCL800 substitution treatments (33%, 66%, and 100% P-BCL800 replacement) significantly improved chili yield, photosynthetic efficiency, soil pH, and soil P availability compared to the traditional phosphate fertilizer treatment. Among these, the 66% substitution treatment achieved the highest yield (382 g) and exhibited the best overall performance. It also enhanced alkaline phosphatase activity and increased microbial diversity, including the relative abundance of Proteobacteria and Actinobacteria. Unlike previous studies that primarily focused on yield and soil nutrients, our findings indicate that P-BCL800 regulates microbial community structure and function in acidic red soil–chili systems. These results highlight key drivers (pH, organic matter, and available P) of sustainable cultivation.

## 1 Introduction

Chili (*Capsicum annuum* L.) holds a distinctive place in global culinary culture. It is a common ingredient that adds unique flavors and textures to various delicacies and is rich in various components beneficial to human health, such as capsaicin, carotene, and vitamin C (Chen et al., 2025). Chili exhibits multiple physiological benefits, such as antioxidant, anti-inflammatory, and anti-cancer properties, and occupies an important position across the food processing, pharmaceutical, healthcare, and condiment industries (Barchenger et al., 2018). It is a major component of diets worldwide, and its commercial demand continues to grow; thus, its planting area and output have concomitantly increased annually (Varghese et al., 2017). According to the Food and Agriculture Organization of the United Nations, annual global chili production exceeds 40 million tons. China ranks among the top countries globally in terms of cultivation area and export volume. Growing market demand for chili has resulted in an increase in the use of chemical fertilizers to increase its yield; however, the long-term and excessive application of fertilizers may result in soil degradation, including soil acidification, nutrient loss, structural damage, and a reduction in beneficial bacteria (Zhu et al., 2005; Khaitov et al., 2019). Therefore, it is necessary to design a fertilization strategy to improve the efficiency of chili cultivation.

From a plant physiology perspective, chili cultivation has relatively high soil nutrient requirements, particularly for phosphorus (P). P is an essential nutrient for plant growth and development because it is a component of many important compounds in plants, including nucleic acids, phospholipids, and ATP. It participates in key physiological processes, such as photosynthesis, respiration, cell division, energy metabolism, and material transportation (Khan et al., 2023). P plays an indispensable role throughout the entire chili growth cycle, contributing to root system development, flower bud differentiation, fruit enlargement, and quality formation. An adequate P supply during the chili flowering and fruiting periods can significantly enhance its fruit setting rate, promote fruit expansion and ripening, and increase fruit weight and quality. In contrast, an insufficient P supply results in slow growth, stunted stature, delayed flowering and fruiting, and dark green and dull leaves, which markedly affects the yield and quality of chili (Khaitov et al., 2019).

In the southern regions of China, chili is primarily cultivated in acidic red soil, which generally contains a low amount of P (total phosphorus: 0.8 g/kg and available phosphorus: 5.5 mg/kg). Under a high-temperature and rainy environment, acidic red soil contains a relatively high content of aluminum and iron hydroxides. The functional groups (–AlOH and –FeOH) existing on the surface have a high chemical affinity for orthophosphate and fix P to form insoluble phosphate, thus reducing P availability (de Campos et al., 2016; Gérard, 2016). Furthermore, the pH of the acidic red soil is relatively low, which is not conducive to soil microorganism activity and further affects P transformation and use. Under these soil conditions, the imbalance between P supply and demand during chili growth is particularly evident. Therefore, farmers must continuously apply P-containing fertilizer to maintain optimal chili yield. Traditional phosphate fertilizers, such as superphosphate, can overcome the P deficiency problem over the short term; however, their utilization rate is generally below 20%. Moreover, excessive application not only significantly increases production costs, which imposes a heavy economic burden on farmers, but also aggravates soil acidification and causes eutrophication of water (Chen et al., 2022) and accelerates the consumption of non-renewable resources, such as phosphate rock (Roy et al., 2017; Nardis et al., 2022). Therefore, it is necessary to increase the bioavailability of P fertilizer and simultaneously improve the acidified soil.

Biochar is a new type of soil conditioner that has shown great application potential in the agricultural field, particularly for improving acidic soil (Ahmed and Hameed, 2020). Several large studies indicate that the application of biochar significantly reduces soil bulk density, increases soil pH, and enhances nutrient availability (Dai et al., 2017; Cooper et al., 2020). Biochar improves acidic soil environments through various pathways: (1) the abundant oxygen-containing functional groups on the biochar surface neutralize soil acidity and reduce the level of exchangeable aluminum (Shetty et al., 2021); (2) the well-developed pore structure of biochar enhances the water-holding capacity and cation exchange capacity (CEC) of the soil, thus promoting nutrient retention (Batista et al., 2018); and (3) biochar acts as a habitat carrier for microorganisms, regulates the activity of soil enzymes, such as phosphatase, and promotes the proliferation of P-solubilizing bacteria (Lehmann et al., 2011; Naeem et al., 2021). However, because of the limited nutrients in biochar, its direct impact on crop yield is not robust (Hussain et al., 2017). Therefore, combining biochar with phosphate fertilizer can simultaneously address biochar’s nutrient deficiency and improve the low bioutilization of phosphate fertilizer, which occurs due to its fixation by soil when used alone.

Studies have indicated that the combination of biochar and fertilizers is more effective at improving soil nutrient utilization rate and crop yield than biochar alone. For example, Wali et al. (2020) prepared biochar by pyrolyzing wheat straw at 350 °C to 400 °C, and then combined it with diammonium phosphate to prepare biochar-based phosphate fertilizer (BPF). Compared with traditional phosphate fertilizer (TPF), a 50% P application rate significantly improved the physiological and chemical characteristics of chickpea plants, resulting in a marked increase in chickpea yield (Pogorzelski et al., 2020) demonstrated that BPF produced with poultry bedding, coffee husks, and superphosphate significantly slowed down P release and increased crop yield in soil with high P fixation. Chintala et al. (2014) reported that P fixation could be reduced through electrostatic adsorption and coordination after loading phosphate onto biochar. The P absorption efficiency in corn roots increased by 40% in their study. Together, the findings indicate that the combined application of biochar and phosphate fertilizer plays a positive role in improving the soil environment and enhancing nutrient utilization efficiency.

Soil microorganisms play an important role in plant growth, soil organic matter mineralization, nutrient cycling, and soil structure improvement. The effect of BPF on soil microorganisms has received extensive attention (Zhang et al., 2019a). Zhang et al. (2019b) examined the responses of fungal communities to biochar, fertilizers, and biochar-based fertilizers in different soils. The results indicated that biochar application significantly affected the alpha -diversity of microorganisms in acidic soils. Xiang et al. (2023) found that the application of low-temperature biochar derived from herbal raw materials significantly increased the diversity of soil microbial communities, directionally enriched functional bacterial communities (e.g., bacillus), and enhanced the activity of soil dehydrogenase by 40%–45%. Moreover, high-temperature pyrolysis biochar (500 °C–600 °C) activated specific bacterial strains, such as Gemmatimonadetes, resulting in the formation of an efficient degradation network, which increased the mineralization rate of soil-refractory carbon by 65% and increased CO_2_ emissions by 93 mg C/kg (Ling et al., 2022). However, studies on biochar-based fertilizers have primarily focused on improving soil nutrients and crop yields. There remains a lack of systematic studies on the influence of BPF on the acidic red soil–chili system, particularly their regulatory effects on the structure and function of soil microbial communities and crop yields. Thus, the underlying mechanism of action of BPF on soil dynamics remains unclear.

Previously, we demonstrated that tobacco stem-based biochar has an enhanced P adsorption capacity because of its *in situ* Ca and Mg content and is capable of improving soil quality and increasing P availability (Tu et al., 2024; Cen et al., 2025); however, its effect on soil microbial communities and broader environmental impact is unclear. In this study, we examined the synergistic effects of BPF on phosphorus retention, availability in the soil, crop phosphorus absorption efficiency, alongside the physical and chemical properties, microbial diversity, and growth of chili in acidic red soil through pot experiments. The results provide a theoretical basis and technical support for the green and efficient cultivation of chili in acidic soils and insights for improving red soil quality and chili production.

## 2 Materials and methods

### 2.1 Materials

Zhujiao No. 15 chili seed was used in this study. It was sown in August and transplanted once the chili seedlings grew to approximately 15 cm. They were transplanted and planted in the Crop Nutrition and Fertilization Research Laboratory of South China Agricultural University in September 2025. Red soil properties are shown in **Supplementary Table 1**. Its organic matter and bioavailable P content were relatively low.

The biochar and the tobacco stem–derived biochar-BPF (P-BCL800) was prepared as described in our previous studies (Tu et al., 2024; Cen et al., 2025). Briefly, tobacco straw was pyrolyzed in a tubular furnace under N_2_ at 800 °C to prepare biochar (termed BCL800), and potassium dihydrogen phosphate was adsorbed. After the adsorption of potassium dihydrogen phosphate, it was vacuumed and dried to produce BPF (termed P-BCL800). The characteristics of biochar are presented in **Supplementary Table 2**. The P content of BPF was calculated based on the adsorption capacity of BCL800. The phosphorus adsorption capacity of biochar was ~25.50 mg/g.

### 2.2 Treatment

To determine the effect of replacing TPF with P-BCL800 on chili growth, soil phosphorus absorption, and soil microorganisms, chili seedlings under the same growth conditions were selected and transplanted into flowerpots that contained 3 kg of red soil. Six different treatment methods were established (**Supplementary Table 3**): blank control (CK), P-BCL800 alone (B), TPF (F), and three alternative treatments, where P-BCL800 replaced 33% (X), 66% (Y), and 100% (Z) of the phosphorus provided by TPFs, respectively. All pots received the same amount of nitrogen, phosphorus pentoxide, and potassium oxide based on 3 kg of soil, whereas the B treatment lacked nitrogen and potassium. The amounts of N, P, and K for each treatment, calculated based on N, P_2_O_5_, and K_2_O, were 0.2, 0.25, and 0.20 g/kg, respectively. The environmental conditions of the experiment were a temperature of 25 °C–30 °C and a relative humidity of 50%–70%. The experiment was conducted from the transplantation of the seedlings to the final harvest (3 months), and systematic sampling of plant and soil parameters was conducted at the end of the experiment.

### 2.3 Measurements of plant growth, soil properties, and nutrient dynamics

This study systematically evaluated the growth performance of chili plants, soil physicochemical properties, key soil enzyme activities, and plant nutrient concentrations. The primary objective was to comprehensively investigate the effects of P-BCL800 on soil phosphorus bioavailability, plant phosphorus acquisition, and soil biochemical transformation processes.

#### 2.3.1 Plant agronomic traits and yield

After planting for 3 months, the agronomic traits of the chili plant, including plant height (PH), stem diameter (SD), shoot fresh weight (SFW), root fresh weight (RFW), fruit yield (YD), SPAD content, and photosynthesis, were measured and analyzed. The PH was measured using a tape measure from the substrate to the growth point. SD was measured using a vernier caliper at a height of 2 cm from the substrate. The chili was harvested when it became shiny and hard, and the entire harvest lasted for 3 months. The entire pot of soil was removed during sample collection, the soil was shaken off, and the rhizosphere soil was used for subsequent soil sample testing. After cleaning the rhizosphere soil and shaking off the water, the fresh weight was measured. SPAD content and photosynthesis were measured in the middle and upper mature leaves using chlorophyll or portable photosynthesis meters, respectively.

#### 2.3.2 Soil physicochemical properties

Electrical conductivity (EC) and pH were measured in a 2.5:1 water/soil mixture using a glass electrode. The alkaline hydrolytic diffusion method was used to determine soil alkaline hydrolytic nitrogen (AHN). Ultraviolet spectrophotometry with ammonium fluoride (NH_4_F) as the extract liquid was used to measure the available phosphorus (AP) (Bornø et al., 2018). Flame photometry with ammonium acetate as an extract liquid was used to measure the rapidly available potassium (RAK) concentration.

#### 2.3.3 Soil enzyme activities

To explore the biotransformation capacity of soil phosphorus, the activities of two pivotal phosphatase enzymes—acid phosphatase (ALP) and alkaline phosphatase (AKP)—were quantified spectrophotometrically using *p*-nitrophenyl phosphate disodium salt (pNPP) as the substrate. For the ALP assay, 1 g (dry weight) of air-dried soil was incubated with acetate buffer (pH 6.5), whereas for the AKP assay, the same soil mass was incubated with glycine–NaOH buffer (pH 11.0); in both cases, pNPP was added as the substrate, and the reactions were conducted at 37 °C for 60 min. The reactions were terminated by adding 0.5 M NaOH, and the liberated *p*-nitrophenol (PNP) was quantified by measuring the absorbance at 400 nm. Enzyme activities were expressed as micromoles of PNP released per gram of dry soil per hour (μmol PNP g^-^¹ h^-^¹).

#### 2.3.4 Plant nutrient content analysis

After the pot experiments were completed, chili seedlings were divided into four parts: root, stem, leaf, and fruit. They were placed into an electric hot air blower box, blanched at 105 °C for 30 min, dried at 75 °C, crushed with a crusher, weighed to 0.10 g, and sterilized with H_2_SO_4_-H_2_O_2_. The total nitrogen (TN) content was determined using the fully automatic Kjeldahl nitrogen determination method. The vanadium- molybdenum-yellow colorimetric method was used to measure total phosphorus (TP), and atomic absorption spectrophotometry was used to measure total potassium (TK).

### 2.4 High-throughput sequencing

The microbial diversity of the rhizosphere soil was detected and analyzed using the Illumina MiSeq platform (Majorbio Bio-Pharm Technology Co., Ltd., Shanghai, China). The composition and diversity of the soil microbial communities were analyzed using 16S rRNA high-throughput sequencing. Total soil DNA was extracted using the MJ-soil Soil DNA Kit (Yuhua Co., Ltd., China) and analyzed by 1% agarose gel electrophoresis by PCR using the following primers: 338F (ACTCCTACGGGAGGCAGCAG) and 806R (GGACTACHVGGGTWTCTAAT) for the 16S rRNA gene. After purification and quantification of the PCR products, a PE library was constructed and sequenced using the Illumina platform. The USEARCH11-uparse algorithm was used for clustering operational taxonomic units (OTUs) and removing chimeras to obtain a representative sequence. For select OTUs or other taxonomic levels with 97% similarity, the alpha-diversity index of different random samples was calculated using Mothur, the beta diversity distance matrix was determined using Qiime (2020.2.0), and analyzed or plotted using the R language (version 3.3.1). Redundancy analysis (RDA). Species Venn plots and community bar charts for soil environmental factors were generated using RDA in the R language vegan package (version 2.4.3). Heatmaps were generated and analyzed using the R language (version 3.3.1) and the Pheatmap package (1.0.8). The LEfSe software was used to conduct species discrimination and to identify species with significant differences in the bacterial community.

### 2.5 Data statistics and analysis

All statistical analyses were conducted using SPSS (version 26.0, IBM Corp.). The significance of differences among treatment mean values for all measured variables (agronomic traits, soil properties, enzyme activities, nutrient contents, and microbial alpha-diversity indices) was determined by one-way analysis of variance. *Post hoc* multiple comparisons were performed using Duncan’s test at a significance level of p < 0.05. Origin 2024 was used to compile the graphs.

## 3 Results

### 3.1 Effect of P-BCL800 on chili agronomic traits

#### 3.1.1 Growth characteristics

The substitution of P-BCL800 for TPF significantly affected the agronomic traits of chili plants. As shown in **Table 1**, **Supplementary Figure 1a**, compared with the F treatment, which contained TPF, partially or completely replacing TPF with P-BCL800 (X, Y, and Z treatments) significantly increased the PH, SD, and RFW of the chili. The effect of the X, Y, and Z treatments on growth was significantly greater than that of the B and F treatments. Of these, Y treatment with 66% P-BCL800 replacing TPF resulted in the greatest PH, whereas X treatment with a substitution ratio of 33% resulted in the best SD and RFW, with no significant differences observed among the X, Y, and Z treatments. For B treatment, the phosphate fertilizer supply method was the same as that of Z. Because of the lack of nitrogen and potassium fertilizers, PH, SD, SFW, and RFW under B treatment were close to those under F treatment but lower than those under Z treatment. Regarding the total output of chili fruits, the final output of chili in each treatment group was ranked as follows: Y > X > Z > F > B > CK. Of these, the output of the Y treatment increased by 93% and 164%, respectively, compared with that of the F and CK treatments. As shown in **Supplementary Figure 1b**, it was also clear that the fruit sizes of the chilies from the Y, X, and Z treatments were significantly greater than those from the B, F, and CK treatments.

**Table 1:** The effects of different treatments on chili growth.

Treatments	Plant height (cm)	Stem diameter (cm)	Shoot fresh weight (g)	Root fresh weight (g)	Yield (g)
CK	21.00 ± 1.33c	4.80 ± 0.10c	3.84 ± 0.24c	2.53 ± 0.36c	57
F	35.14 ± 1.68b	5.73 ± 0.22b	8.06 ± 0.17ab	4.71 ± 0.42b	198
B	35.18 ± 3.19b	6.08 ± 0.12ab	6.65 ± 0.36b	4.78 ± 0.32b	147
X	51.26 ± 2.49a	6.60 ± 0.22a	10.27 ± 0.54a	8.14 ± 0.38a	344
Y	54.4 ± 1.79a	6.50 ± 0.20a	9.87 ± 0.53a	8.48 ± 0.25a	382
Z	53.44 ± 1.44a	6.18 ± 0.15ab	8.56 ± 1.64ab	8.10 ± 0.34a	321

Different lowercase letters represent significant differences between different treatments at P < 0.05.

#### 3.1.2 Photosynthetic characteristics

Photosynthetic parameters in mature chili leaves were significantly enhanced by P-BCL800 application (**Table 2**). The chlorophyll content (SPAD content), net photosynthetic rate (Pn), and stomatal conductance (Gs) were ranked as follows: Y > Z > X > B > F > CK. There was no significant difference among the X, Y, and Z treatments (P < 0.05); however, they were significantly better compared with the B, F, and CK treatments. This indicates that the application of P-BCL800 significantly improved the photosynthetic characteristics of the chili plant. However, changes in intercellular CO_2_ concentration (Ci) exhibited the opposite tendency, which was in the order: CK > B > F > Y > Z > X. In addition, only the transpiration rate (Tr) resulting from Y treatment showed a significant difference with CK treatment, but no significant difference for most other treatments. This indicates that the effect of P-BCL800 on the Tr of chili in the present study was relatively low; therefore, the higher Tr observed with Y treatment may result from its greater stomatal conductance.

**Table 2:** Photosynthetic characteristics of chili under different treatments.

Treatments	SPAD	Net photosynthetic rate (μmol·m^−2^·s^−1^)	Stomatal conductance (mol·m^−2^·s^−1^)	Intercellular CO_2_ concentration (μmol·mol^−1^)	Transpiration rate (mmol·m^−2^·s^−1^)
CK	36.32 ± 1.52c	2.53 ± 0.36c	0.061 ± 0.005b	261.47 ± 6.74a	2.02 ± 0.06b
F	41.48 ± 1.72bc	4.71 ± 0.42b	0.077 ± 0.008b	183.25 ± 6.50c	2.45 ± 0.25ab
B	45.1 ± 1.20b	4.78 ± 0.32b	0.082 ± 0.010b	204.17 ± 4.88b	2.64 ± 0.22ab
X	53.88 ± 2.33a	8.14 ± 0.38a	0.118 ± 0.013a	155.19 ± 8.04e	2.49 ± 0.26ab
Y	55.86 ± 1.84a	8.48 ± 0.25a	0.124 ± 0.012a	175.18 ± 5.72cd	2.72 ± 0.15a
Z	54.76 ± 2.67a	8.10 ± 0.34a	0.117 ± 0.012a	159.25 ± 6.32de	2.16 ± 0.19ab

Different lowercase letters represent significant differences between different treatments at P < 0.05.

#### 3.1.3 Nutrient accumulation

**Figure 1:**
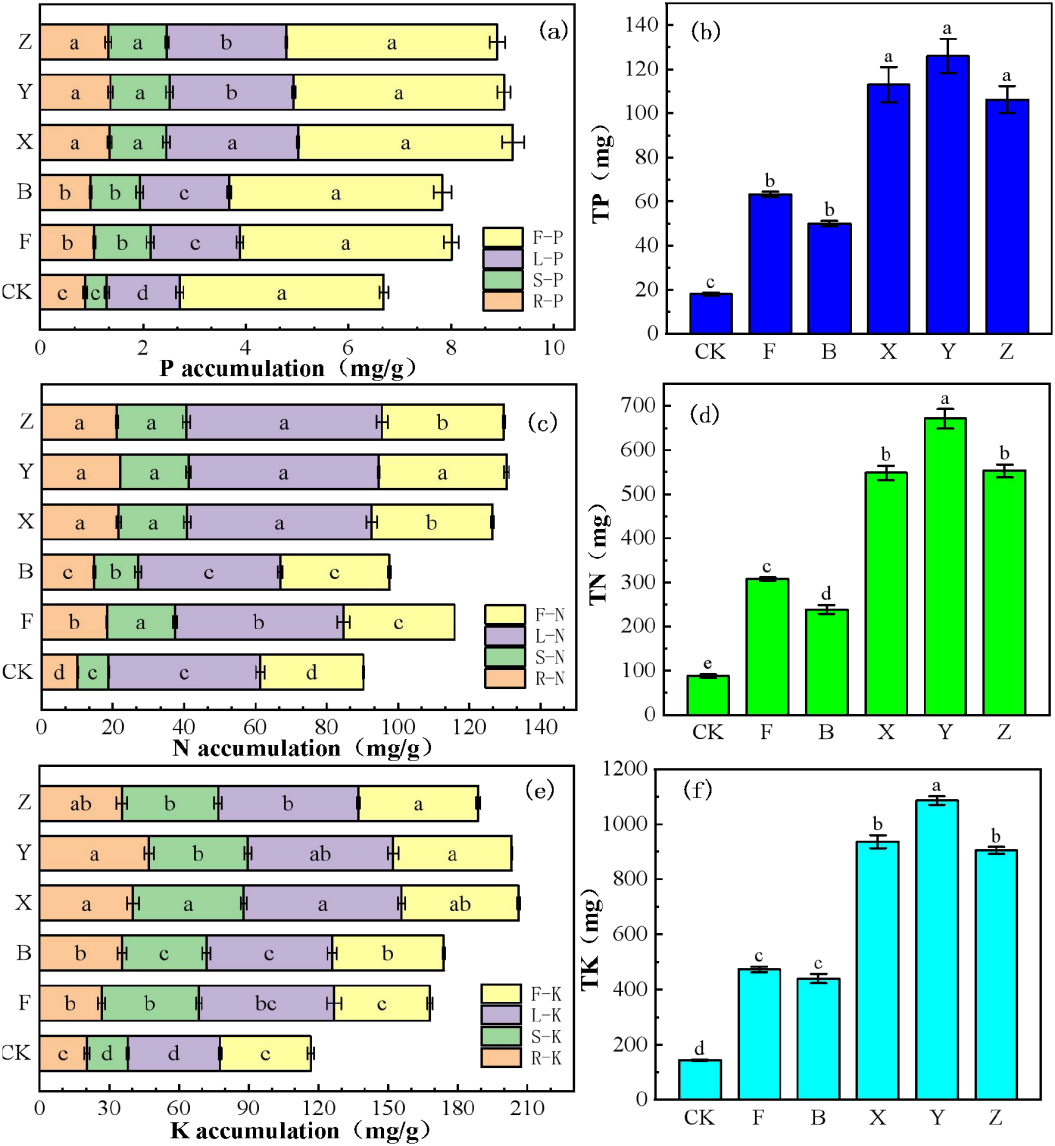
Accumulation of P **(a)**, N **(c)**, and K **(e)** in the roots, stems, leaves, and fruits of chili plant under various treatment conditions. TP **(b)**, TN **(d)**, and TK **(f)** in chili. Different letters indicate significant difference (Tukey’s HSD test, P < 0.05; n = 5).

Partially or completely replacing TPF with P-BCL800 (X, Y, and Z treatments) markedly increased P in the roots, stems, leaves, and fruits of the plants compared with the controls (F, CK, and B treatment, **Figures 1a, b**). The highest value for TP accumulation was observed following Y treatment (66% P-BCL800), which increased by 30%, 40%, and 39% in the root, stem, and leaf compared with F treatment; however, it showed no significant difference in the fruit, and no significant differences were observed for X, Y, and Z treatments. A similar tendency was observed for N (**Figures 1c, d**), TN, K, and TK (**Figures 1e, f**) accumulation in the chili. The highest value for N and K accumulation was observed for the Y treatments compared with the F treatment. N accumulation increased by 21%, 13%, and 15% in the root, leaf, and fruit, respectively, whereas K accumulation increased by 28%, 14%, 17%, and 22% in the root, stem, leaf, and fruit parts. No significant difference was observed following X, Y, and Z treatments.

### 3.2 Effect of P-BCL800 on soil properties

#### 3.2.1 Physical and chemical properties

The physical and chemical properties of the soil before and after chili planting are shown in **Supplementary Tables 1**, **3**. The soil pH increased with an increased proportion of P-BCL800. However, the highest pH was observed following B treatment with a single application of P-BCL800, at 7.09, which was 23% higher than that observed for F treatment. Moreover, the organic matter (OMC) and EC of the soil were ranked as follows: Z > Y > X > F > B > CK, with significant differences among all treatments. The highest OMC and EC were observed for Z treatment at 32.65 mg/g and 281.2 µS/cm, respectively, and increased by 354.10% and 31.16% compared with F treatment. It is worth to mention that, soil EC under the highest P-BCL800 rate (Treatment Z) was 281.2 ± 9.35 µS/cm, remaining below the conventional salinity threshold of 0.8 mS/cm for non-saline conditions (Salinas et al., 2023).

The AHN content following B, X, Y, and Z treatments was slightly higher than that of CK and B treatments; however, no significant difference was observed following F treatment, indicating that biochar caused little change in soil N. The AP resulting from the B, X, Y, and Z treatments increased by 154%, 101%, 134%, and 151% (P < 0.05) respectively, compared with the F treatment. In addition, rapidly available potassium (RAK), exchangeable Ca^2+^ (E_Ca_), and exchangeable Mg^2+^ (E_Mg_) were also increased following P-BCL800 application. Taken together, the application of P-BCL800 enhances soil pH and EC values, affects nutrient availability, and improves overall soil fertility. The greater the application amount, the more significant the improvement effect.

#### 3.2.2 Phosphatase activity

**Figure 2:**
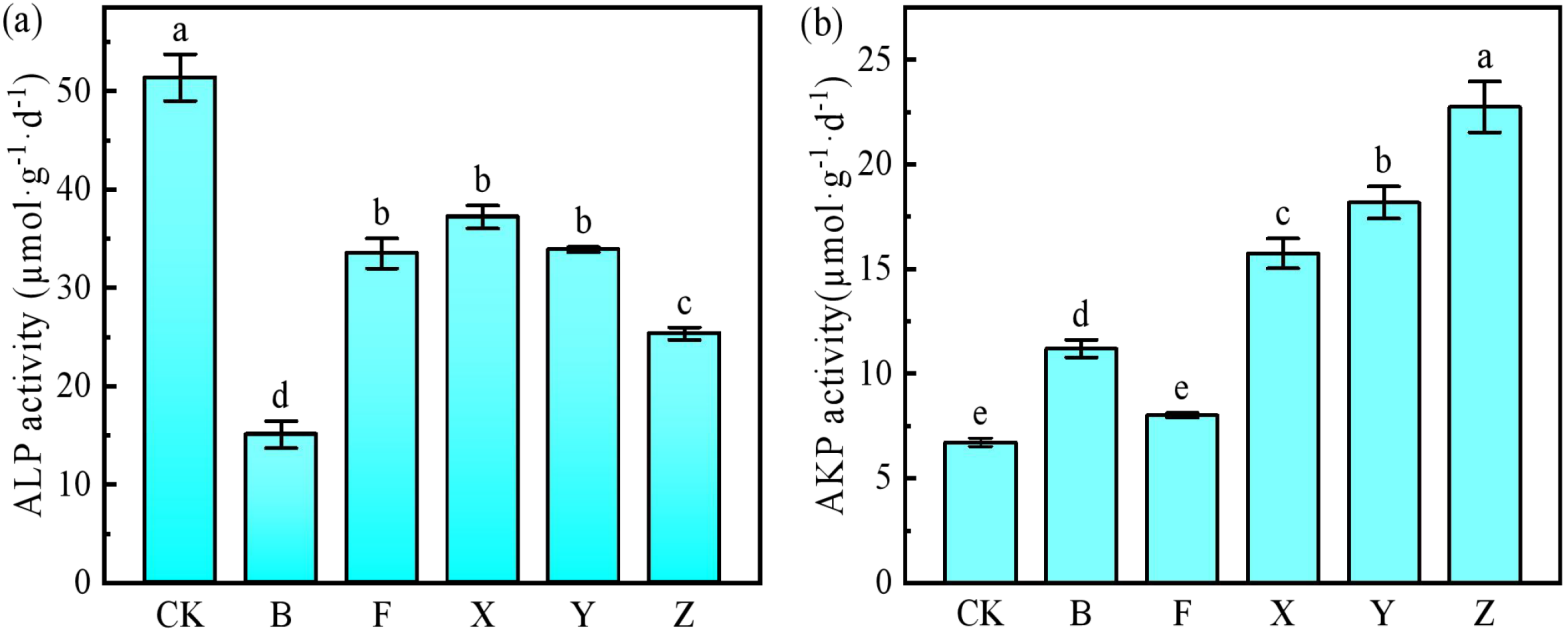
Acid phosphatase (ALP) **(a)** and alkaline phosphatase (AKP) **(b)** activities under various treatments. Different letters indicate significant difference (Tukey’s HSD test, P < 0.05; n = 5).

Acid phosphatase (ALP) activity decreased after the application of phosphate fertilizers, which was negatively correlated with the AP content in the soil (**Figure 2**, **Table 3**); however, AKP activity exhibited an opposite tendency. It was much lower in the CK and F treatments because of the lower soil pH. It increased after the application of P-BCL800, and its activity positively correlated with the AP content in the soil. Although B treatment was also fully applied with P-BCL800, because of the deficiency of N and K, the ALP and AKP activities following B treatment exhibited a different pattern compared with the other treatments.

**Table 3:** Soil physical and chemical properties associated with different treatments after planting.

Parameters	CK	F	B	X	Y	Z
pH	5.54 ± 0.01d	5.35 ± 0.06e	7.09 ± 0.02a	5.81 ± 0.12d	6.39 ± 0.06c	6.60 ± 0.06b
EC (µS/cm)	145.2 ± 4.99e	214.4 ± 7.45c	184.8 ± 6.11d	251.2 ± 7.14b	269.6 ± 5.33ab	281.2 ± 9.35a
OMC (mg/g)	5.62 ± 1.01d	7.19 ± 0.66d	31.22 ± 0.97a	12.19 ± 0.75c	20.97 ± 0.87b	32.65 ± 1.99a
AHN	28.00 ± 0.49c	36.12 ± 1.26a	32.20 ± 1.40b	38.36 ± 0.71a	38.92 ± 0.85a	39.06 ± 0.87a
AP (mg/kg)	0.37 ± 0.03e	3.21 ± 0.51d	8.15 ± 1.08a	6.47 ± 0.72c	7.51 ± 0.79ab	8.06 ± 0.64a
RAK (mg/kg)	71.52 ± 3.42d	150.20 ± 3.62b	105.80 ± 2.57c	154.01 ± 2.18ab	157.92 ± 2.95ab	162.56 ± 3.47a
E_Ca_ (mg/kg)	102.78 ± 2.38d	95.84 ± 2.35d	184.51 ± 3.95a	137.57 ± 3.06c	150.43 ± 2.86b	153.59 ± 3.64b
E_Mg_ (mg/kg)	17.45 ± 0.71d	16.95 ± 0.55d	26.29 ± 0.29a	21.21 ± 0.30c	24.12 ± 0.20b	25.63 ± 0.51a

Different lowercase letters represent significant differences between different treatments at P < 0.05.

#### 3.2.3 Correlation coefficients among soil physicochemical properties and chili agronomic traits

**Figure 3:**
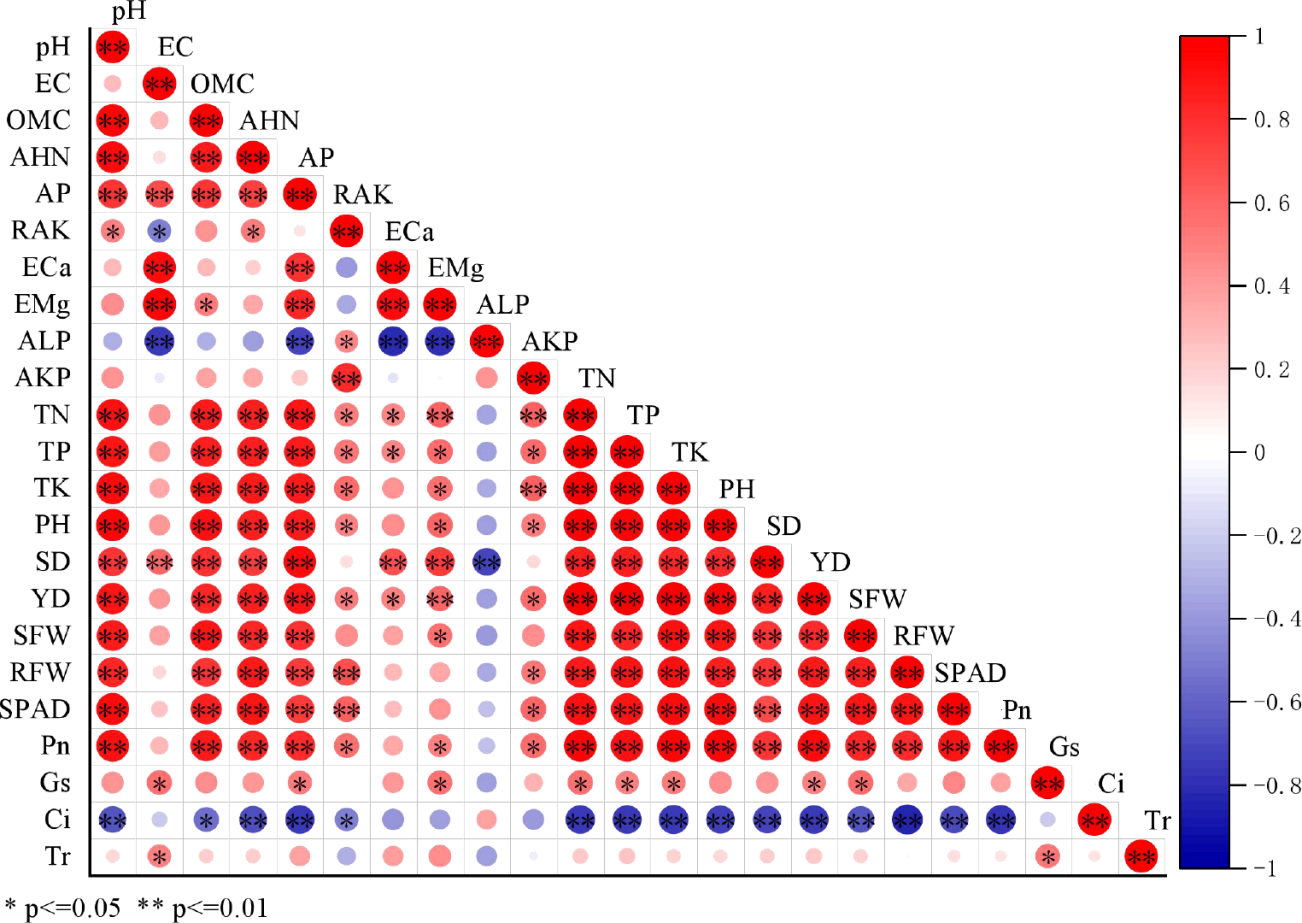
Correlation coefficients between soil physicochemical properties and chili agronomic traits.

Regarding the color scale on the right side of **Figure 3**, red represents a positive correlation, and blue indicates a negative correlation. The depth of the color reflects the strength of the correlation. Significant positive correlations (P ≤ 0.01) were detected between key soil properties (pH, OMC, AHN, and AP) and chili growth parameters (PH, SD, SFW, RFW, and YD) as well as nutrient content (TN, TP, and TK) (**Figure 3**). A strong positive correlation was also found between chlorophyll content (SPAD) and Pn, and a negative correlation was found only in ALP and Ci with other parameters.

### 3.3 Effect of P-BCL800 on soil microbial communities

#### 3.3.1 Venn diagram analysis

**Figure 4:**
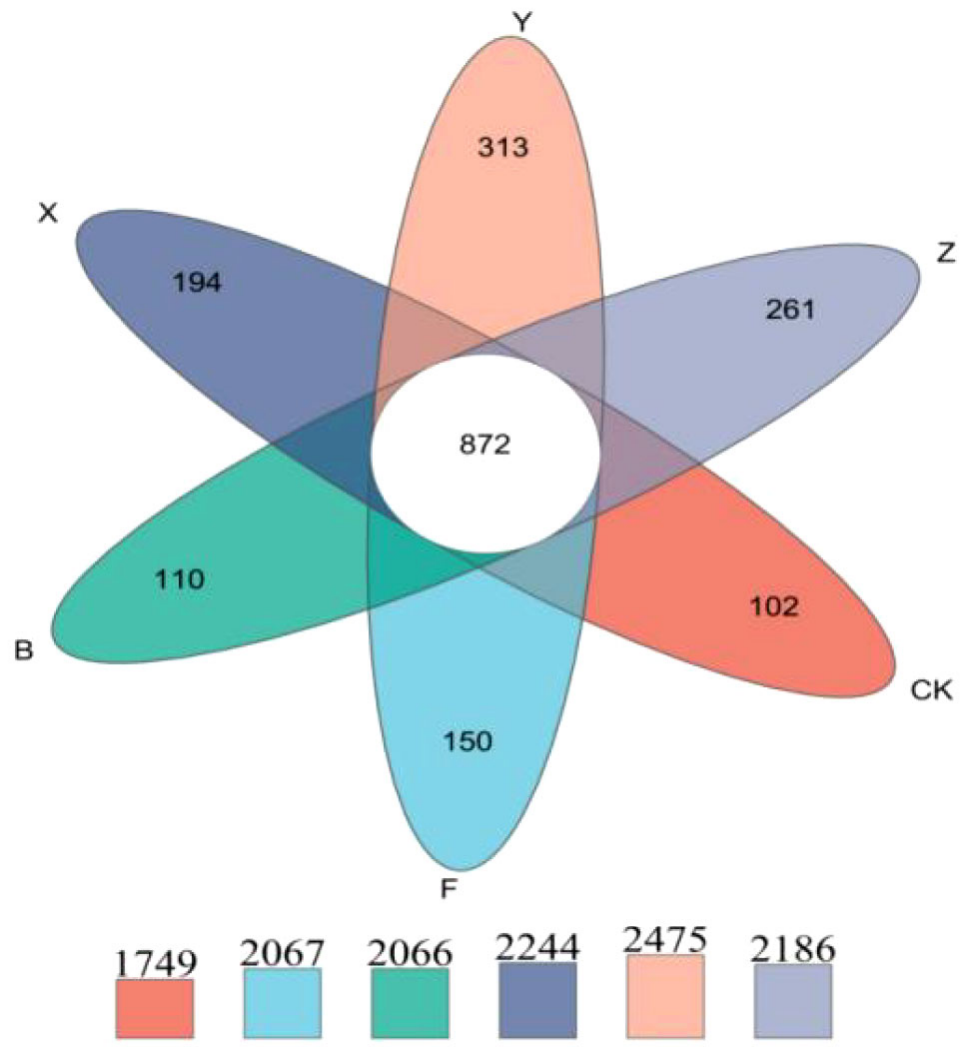
Venn diagram of the unique species at the phylum level under different treatments.

The Venn diagram in **Figure 4** indicated that the core microbiome OTU number was 872, whereas the unique OTU numbers for the CK, F, B, X, Y, and Z treatments were 102, 150, 110, 194, 313, and 261, and the total OTU numbers were 1749, 2064, 2066, 2244, 2475, and 2186, respectively. P-BCL800 significantly affected the unique OTU numbers, which increased by 30%, 108%, and 74% for the X, Y, and Z treatments compared with those of the F treatment, and the total OTU numbers increased by 8.6%, 19.7%, and 5.8%, respectively, indicating a marked enrichment of microbial diversity.

#### 3.3.2 Soil microbial alpha diversity

**Figure 5:**
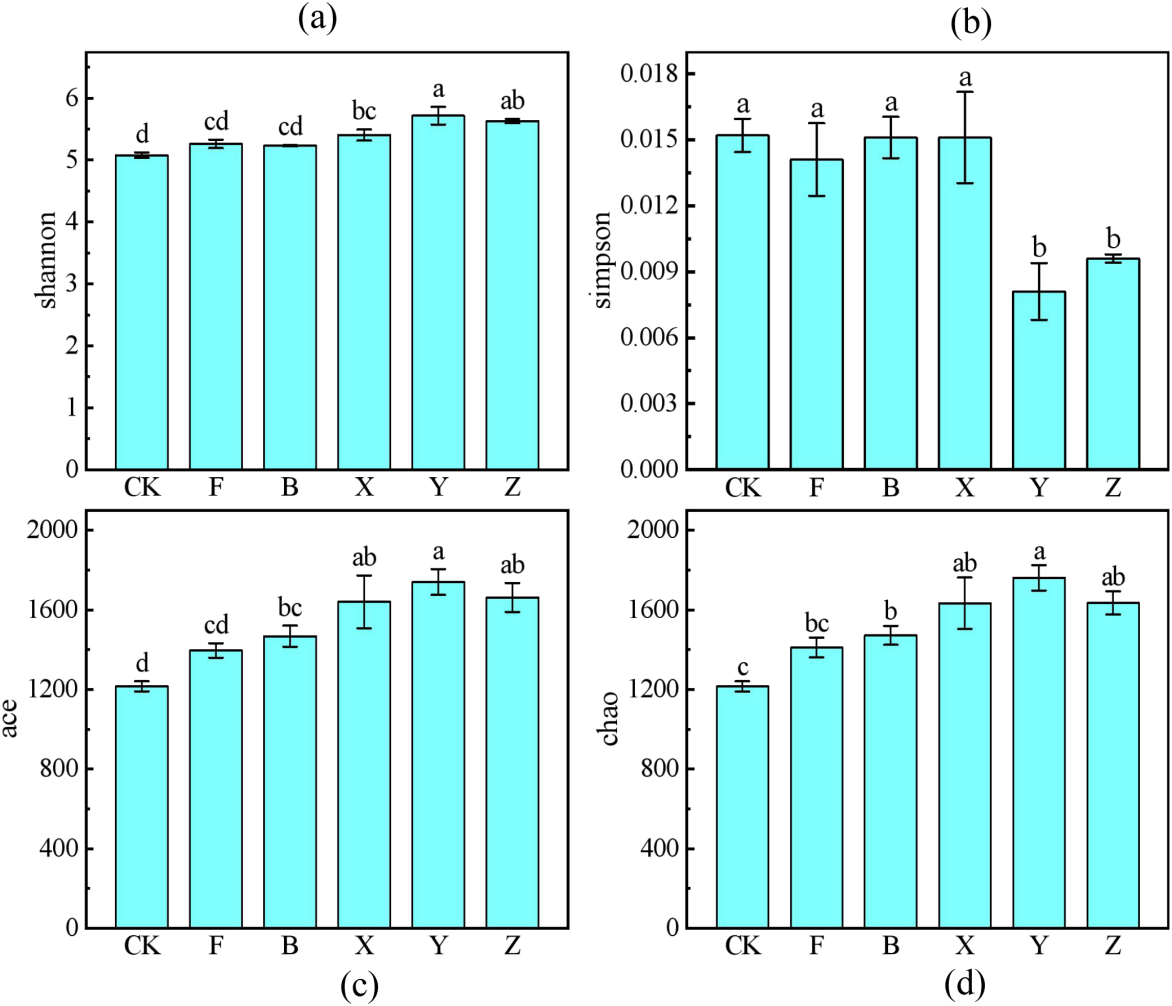
Soil microbial alpha diversity under various treatments. Shannon index **(a)**, Simpson index **(b)**, Ace index **(c)**, and Chao index **(d)**. Different letters indicate significant difference (Tukey’s HSD test, P < 0.05; n = 5).

Soil microbial alpha diversity is used to assess the richness and diversity of microbial communities in environmental samples. The alpha-diversity indices shown in **Figure 5** were calculated using 16S rRNA gene sequence data at the OTU level. Significant differences in microbial diversity were observed among all treatments. The Shannon index is used to measure the unpredictability of the occurrence of individual species in a community. Y treatment yielded the highest Shannon indices, followed by Z > X > F > B > CK; however, no significant difference was evident among the X, Y, and Z treatments, indicating that the application of P-BCL800 significantly enhanced soil microbial diversity. The Simpson index value ranges from 0 to 1 and is an indicator of the concentration and evenness of species within a microbial community. Simpson indices for the CK, F, B, and X treatments showed no significant difference but were lower than those for the Y and X treatments, indicating a more homogeneous microbial community following the Y and Z treatments. Chao1 and Ace indices are often used to reflect the richness of a species.

#### 3.3.3 Soil microbial beta diversity

**Figure 6:**
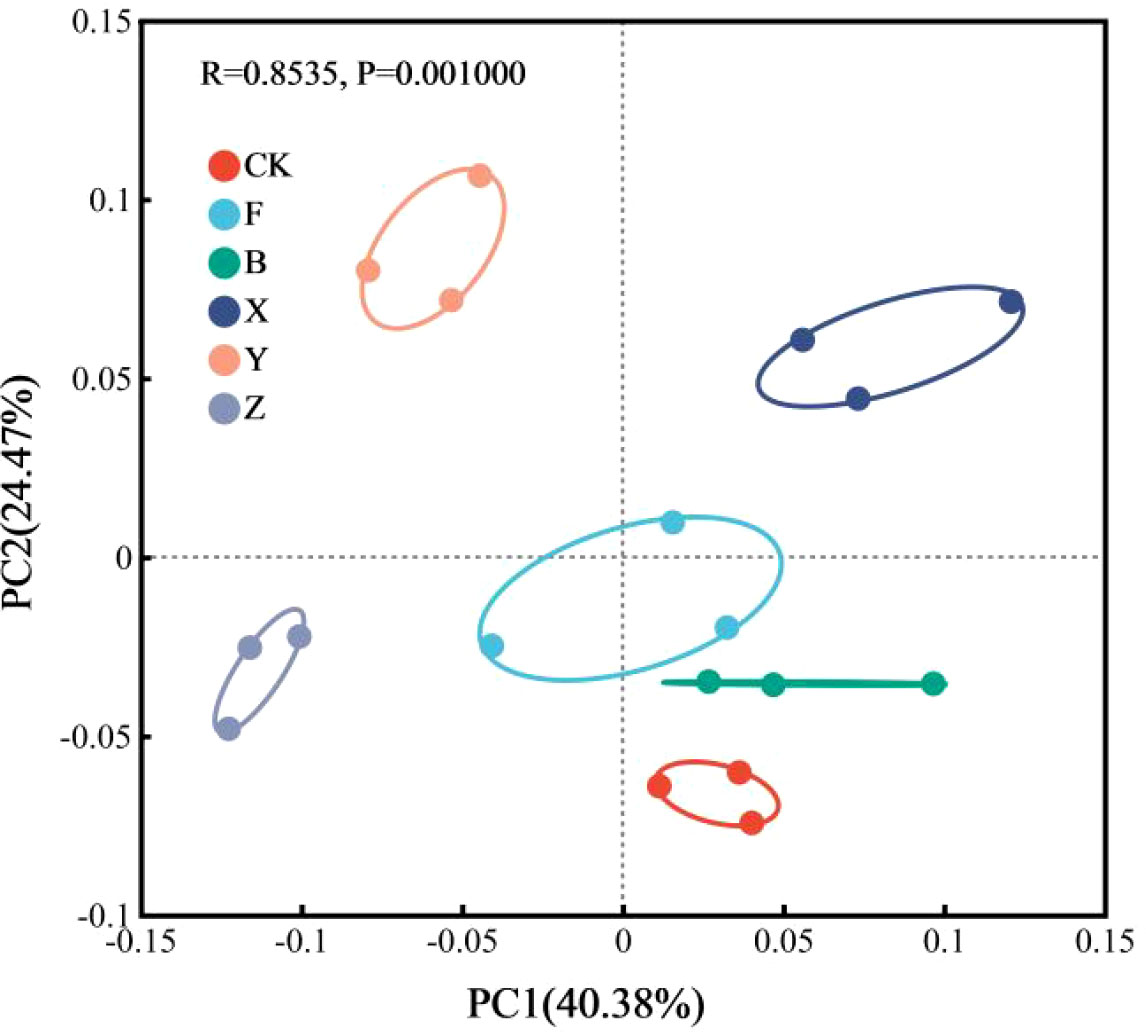
Principal coordinate analysis under different treatments.

Principal coordinate analysis based on Bray–Curtis distances revealed a clear separation of microbial community structures among treatments (**Figure 6**). Differences in CK, B, and F treatments were relatively small, whereas under X, Y, and Z treatments, the composition was distributed throughout the remaining three quadrants and relatively far apart, indicating that the application of P-BCL800 significantly changed the community composition.

#### 3.3.4 Soil microbial community

**Figure 7:**
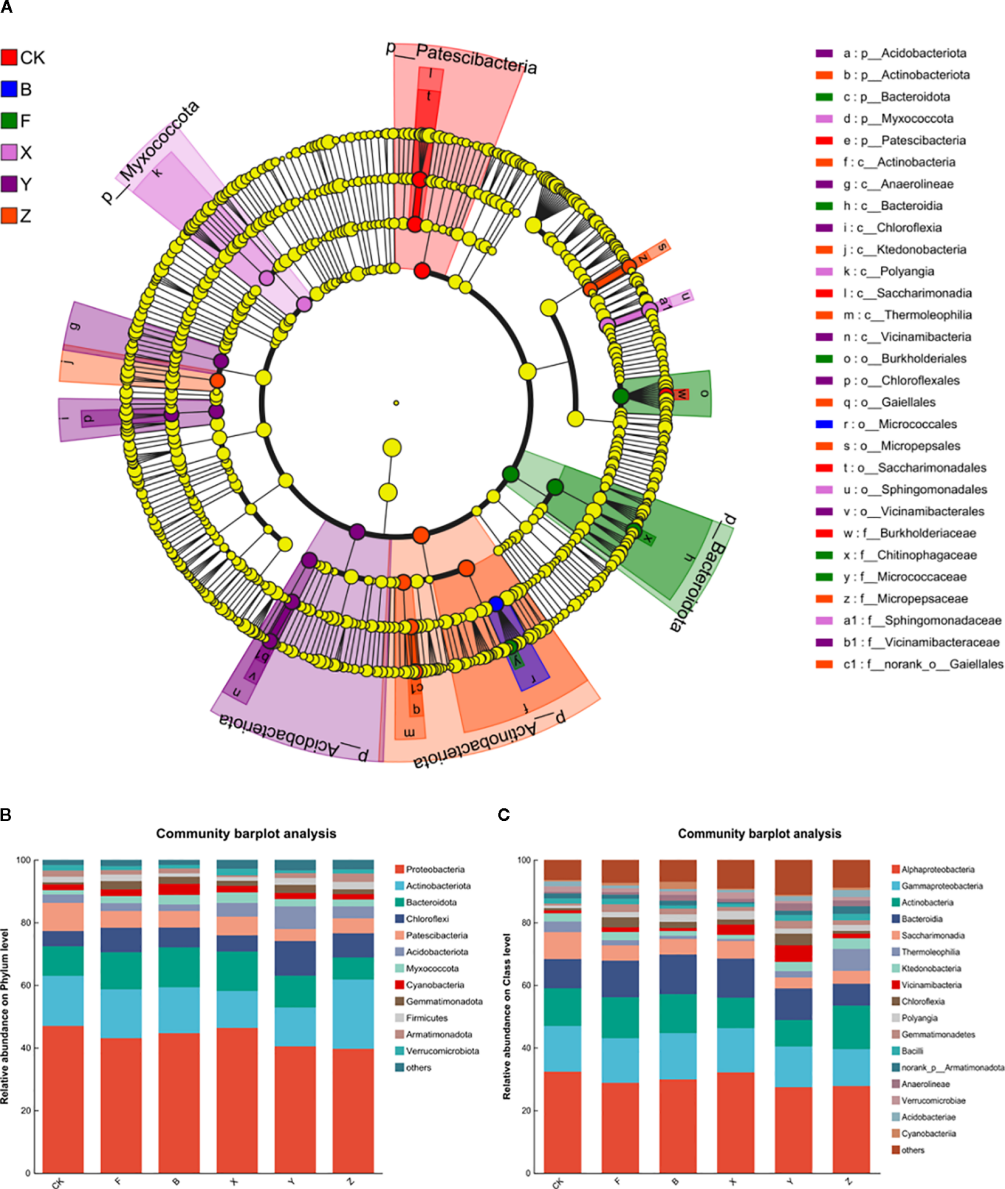
Linear discriminant analysis effect size (LEfSe) analysis **(a)**, and community composition and relative abundance of bacterial phylum **(b)** and class **(c)**. Colored shadows represent the tendency of the significantly different taxonomic groups. Each colored point has a LDA, linear discriminant analysis score of effect size, and only the taxonomic groups that meet an LDA significance threshold >4 is displayed.

The linear discriminant analysis effect size (LEfSe) method was used to identify significant differences in bacterial community abundance under all treatments. Taxonomic groups showing significant differences in abundance are indicated by colored dots and represent the phylum, class, family, and genus levels from the center outward. As shown in **Figure 7a** and S2, the relative abundance of Acidobacteria following Y treatment and Actinobacteria following Z treatment markedly changed. The predominant microbial communities after CK, B, F, and X treatments were *Patescibacteria, Bacteroidota, Myxococcota*, and *Actinobacteria.*

The soil bacterial communities were dominated by Proteobacteria, Actinobacteria, Bacteroidota, and Chloroflexi at the phylum level (**Figure 7b**). Their relative abundances across treatments ranged from 39.67%–46.95%, 11.78%–22.10%, 7.05%–12.74%, and 4.88%–11.07%, respectively. Compared with F treatment, the relative abundance of Chloroflexi following Y treatment, Proteobacteria following X treatment, and Actinobacteria following Z treatment was markedly increased, reflecting the differential impact of P-BCL800 on the microbial groups. At the class level, Alphaproteobacteria, Gammaproteabacteria, Actinobacteria, and Bacteroidia were dominant under all treatments, with their relative abundance ranging from 27.40% to 32.38%, 11.75% to 14.76%, 8.43% to 13.89%, and 7.00% to 12.67%, respectively.

#### 3.3.5 Correlation between soil microbial community and soil properties

As shown in **Table 4**, soil microbial diversity indices responded to soil indicators. The Shannon index significantly responded to EC and OMC (P < 0.01) as well as to AP and RAK (P < 0.05). The Simpson index only significantly responded to EC and OMC. The Ace index significantly responded to AKP and the Chao index to EC, OMC, AP, and RAK. These results indicate that soil microbial diversity and richness are affected by EC, OMC, AP, and RAK. The changes observed in soil EC, OMC, AP, and RAK were attributed to P-BCL800 application. Thus, P-BCL800 alters microbial diversity and richness by influencing the physical and chemical properties of the soil. Moreover, soil with higher EC, OMC, AP, and RAK exhibited increased microbial diversity and richness.

**Table 4:** Correlation between soil physical and chemical properties and microbial diversity.

Indexs	pH	EC	OMC	AHN	AP	RAK	E_Ca_	E_Mg_	ALP	AKP
shannon	0.333	0.643**	0.692**	0.336	0.437*	0.439*	0.208	0.355	0.114	0.445*
simpson	−0.377	−0.435*	−0.530**	−0.141	−0.358	−0.264	−0.218	−0.329	−0.044	−0.237
ace	0.334	0.466*	0.484*	0.254	0.468*	0.438*	0.297	0.353	0.125	0.574**
chao	0.328	0.500*	0.515**	0.292	0.469*	0.481*	0.290	0.359	0.109	0.558**

*P < 0.05, **P < 0.01.

**Figure 8:**
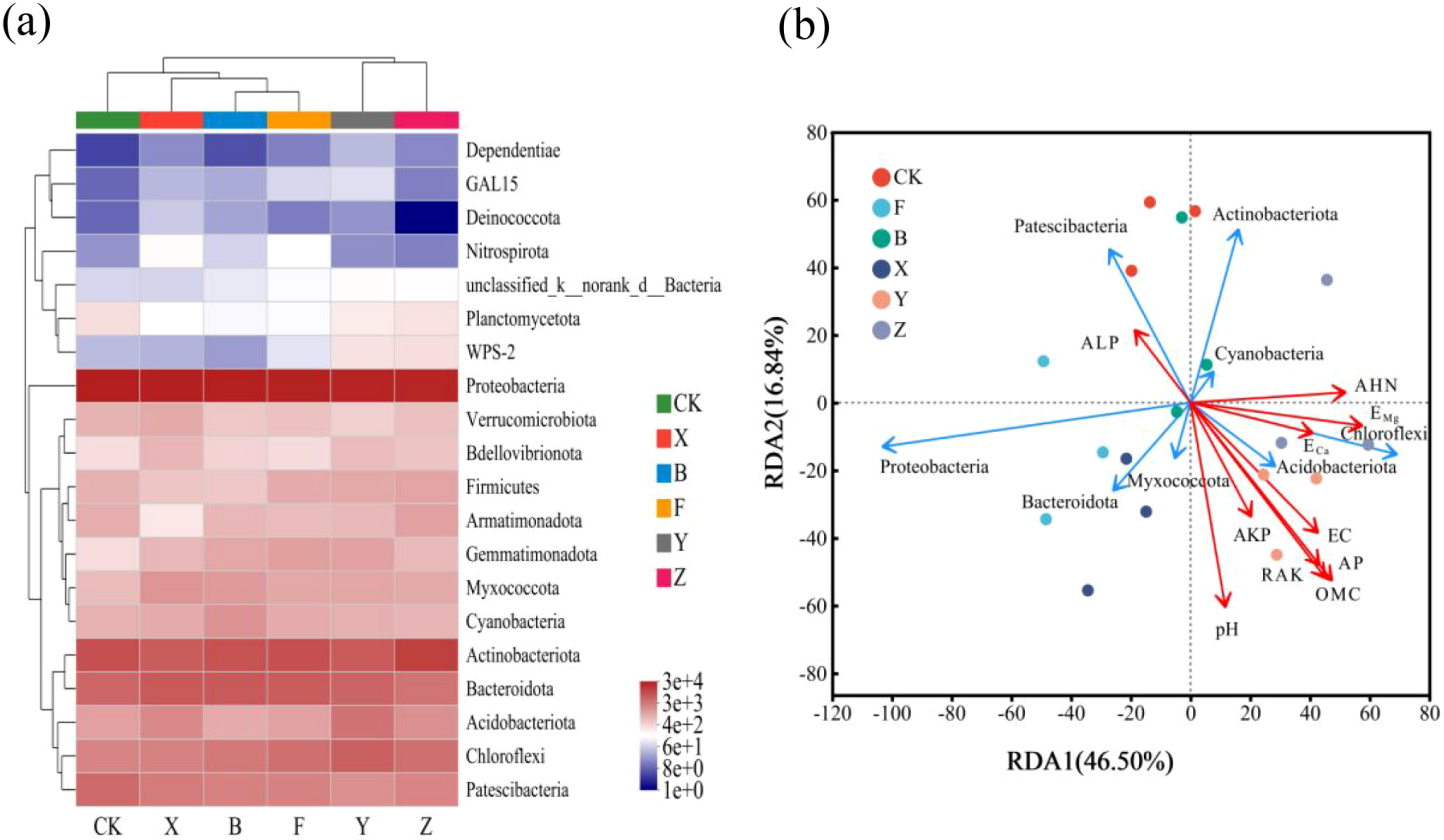
**(a)** Heatmap of the top 20 species with the highest abundance at the phylum; **(b)** RDA, Redundancy analysiscorrelation between soil microbial community structure and soil nutrients.

**Figure 8a** presents the heatmap of the top 20 species with the highest abundance at the phylum level under various treatments. Compared with the F treatment, the Y and Z treatments resulted in a similar community structure, which was significantly different from that of the CK, B, F, and X treatments. The growth of Planctomycetota, WPS-2, and Proteobacteria in Y and Z treatments was enhanced, whereas Nitrospirota and Deinococcota were inhibited. Furthermore, Patescibacteria, Chloroflexi, Acidobacteria, Bacteroidota, and Actinobacteria were the dominant bacteria under all treatments. The addition of P-BCL800 increased the relative abundance of Acidobacteria, Chloroflex, and Actinobacteria but reduced that of Nitrospirota and Deinococcota. This suggests that P-BCL800 can enhance beneficial microbial populations that may contribute to soil fertility.

Based on the microbial diversity analysis at the phylum level, the top eight abundant species were identified. The results of the RDA between microbial diversity and soil environmental factors at the phylum level are shown in **Figure 8b**. The soil rhizosphere bacterial communities for the X, Y, and Z treatments were primarily distributed in the first, third, and fourth quadrants, which were positively correlated with the physical and chemical properties of the soil, indicating a significant effect on the bacterial community structure following the addition of P-BCL800. Particularly, pH, EC, OMC, AHN, AP, E_Ca_, E_Mg_, and AKP were positively correlated with Chloroflexi and Acidobacteria but negatively correlated with Patescibacteria. Moreover, ALP was negatively correlated with other soil indicators. The top three environmental factors influencing soil microbial communities following the addition of P-BCL800 were OMC, pH, and AP.

## 4 Discussion

This study provides novel evidence that tobacco stem–derived biochar-BPF (P-BCL800) can improve chili productivity and soil fertility in acidic red soil and significantly reshape the structure and function of the soil microbial community. Although previous research on biochar-based fertilizers primarily focused on crop yield and nutrient availability, our study systematically linked P-BCL800 application to specific changes in microbial diversity, composition, and functional potential, thereby identifying soil pH, organic matter, and AP as key mediating factors.

### 4.1 P-BCL800 improve chili agronomic traits

This study demonstrated that the PH, SD, and RFW of chili significantly increased as partially or completely replacing TPF with P-BCL800 (X, Y, and Z treatment). This result was consistent with those obtained by Wali et al. (2020), who reported that P-enriched biochar significantly improved crop growth, yield, and nodulation. P promotes early root development, and as an important component of nucleic acids, it plays a vital role in the growth and development of plants (Dotaniya et al., 2014). The improvement in PH, SD, SFW, and RFW of chili was likely the result of the high availability of P throughout the crop growth cycle. Moreover, according to Xiao et al., biochar with wide pores could improve soil nutrient penetration and water-holding capacity (Lehmann et al., 2003; Xiao and Pignatello, 2016), soil CEC, and soil microbial activity (Liang et al., 2006; Deng et al., 2024). Thus, the enhanced physiochemical and biological properties of soil may also lead to improved chili growth.

The fruit sizes and final output of chili were also increased with the use of P-BCL800. Biochar was previously shown to be capable of directly or indirectly enhancing nutrient use efficiency by increasing organic carbon in the soil (Li et al., 2022) and decreasing nutrient leaching (Yuan et al., 2016). The application of biochar increased crop yield because of the enhanced P, nitrogen, and potassium utilization efficiency (Arif et al., 2017; Zhang et al., 2020; Murtaza et al., 2021). In other words, biochar enhanced the biological, physical, and chemical characteristics of the soil, including the pH and CEC, which resulted in greater agricultural productivity (Lu et al., 2022). P-BCL800 comprises biochar and chemical fertilizers, of which biochar has a rich pore structure and strong ion adsorption capacity. It can not only improve soil acidity but also reduce the leaching and fixation loss of nutrient and increase the soil’s mineral absorption capacity, thus meeting the nutrient requirements of chili throughout their growth period (Zhang et al., 2017).

Photosynthetic characteristics such as SPAD content, Pn, and Gs in chili improved significantly, but Ci decreased and Tr showed no obvious difference with the application of P-BCL800. The increase in SPAD content was likely due to the impression of P-BCL800 on plant nutritional status (Lusiba et al., 2017), as the enhanced uptake of C, N, and P, particularly the improved N content, had a remarkable effect on chlorophyll content (Martínez and Guiamet, 2004; Agegnehu et al., 2015). However, because of the deficiency of other nutrient elements, SPAD content, Pn, and Gs, B treatment were all close to those in F. Similarly, the improved net photosynthetic rate and stomatal conductance following P-BCL800 application were likely the result of the high moisture content and P availability in the soil (Ulyett et al., 2014). The moisture and P content play important roles in plant growth and morphology (Ahmad et al., 2020), and biochar improved soil moisture retention and P availability (Glaser et al., 2002; Shi et al., 2023). Moreover, previous studies demonstrated that biochar and P may promote stomatal conductance, resulting in a marked flow of CO_2_ to the leaf mesophyll cells, resulting in a higher net photosynthesis rate (Ye et al., 2016). The decrease in Ci may be the result of increased SPAD values by P-BCL800, delayed leaf senescence, and enhanced photosynthetic efficiencies (Liu et al., 2022a). As CO_2_ is the raw material of photosynthesis, increased photosynthesis enables leaves to increase intercellular CO_2_ utilization efficiency, resulting in a decrease in Ci.

The markedly increased P in the roots, stems, leaves, and fruits of the plants in X, Y, and Z treatments compared with the controls (F, CK, and B treatment), demonstrating enhanced P uptake by P-BCL800 application (Joseph et al., 2021). Previous studies indicate that biochar enhanced P retention while minimizing P leaching through adsorption and desorption processes. Increased biological activity for P solubilization provided a continuous source of P for the growth of the chili plant, along with increased P accumulation in the chili (Ghodszad et al., 2022; Shi et al., 2023). Moreover, BPF improved soil nutrient supply status by activating soil nutrients, enhancing soil fertility, reducing the disadvantages of poor soil compaction and permeability, as well as the decline in organic matter. This promoted the growth of the chili root systems and thereby ensured the absorption and utilization of P (Pandian et al., 2024). The accumulation of N, TN, K, and TK in chili indicated that P-BCL800 also improves the uptake of N and K (Chen et al., 2023; Ahmed et al., 2024). One possible reason is that the application of P-BCL800 may enhance P availability, which in turn facilitates the uptake of N along with other nutrients (Zubillaga et al., 2002; Balai et al., 2017). The application of P-BCL800 may improve the physical and chemical properties of the soil and increase the availability of soil nutrients. The large specific surface area and rich pore structure of biochar effectively reduce the leaching of soil nutrients, enhance the soil adsorption capacity of the nutrients, and promote the absorption of N, P, and K by the chili plant (Zheng et al., 2020).

### 4.2 P-BCL800 enhances the physicochemical properties of soil

Physical and chemical properties of the soil are of great importance to plant and are affected by the application of P-BCL800. Because of the high-pH biochar in BPF and its effect on raising soil pH and buffering acidity (Lu et al., 2022; Tu et al., 2024), the soil pH increased with an increased proportion of P-BCL800. However, B treatment got the highest pH, this may be attributed to the lack of N nutrition. In general, N was applied in the form of urea, which was hydrolyzed into NH_4_^+^ and absorbed by the plants directly or converted into NO_3_^−^ through nitrification. The absorption and leaching of NO_3_^−^ were accompanied by the production of H^+^ and contributed to soil acidification (Tong and Xu, 2012; Qian and Chen, 2013). Moreover, TPF and P-BCL800 were all capable of releasing loosely bound nutrients/elements into the soil; however, because of the highly recalcitrant nature, and high K, Ca, and Mg content in biochar used in the present study (Warnock et al., 2007; Kuzyakov et al., 2009; Tu et al., 2024), soil EC and OMC were significantly enhanced by P-BCL800 application, which is consistent with the results of previous studies (Atkinson et al., 2010; Ahmad et al., 2012; Shaaban et al., 2018). In addition, the application of P-BCL800 increased AP, E_Ca_, and E_Mg_ in soil, which was similar to the results of Zhang et al (Zhang et al., 2019a). This occurred because P-BCL800 acts as a slow-release fertilizer and increases the P bioavailability in the soil. Biochar also helps capture P complexing metallic ions and affects P adsorption/desorption equilibrium, thus reducing P fixation (Mendes et al., 2014) and influencing P dynamics in the soil (Gao et al., 2016). Taken together, the application of P-BCL800 enhances soil pH and EC values, affects nutrient availability, and improves overall soil fertility. The greater the application amount, the more significant the improvement effect.

Acid phosphatase (ALP) and alkaline phosphatase (AKP) activities are important for promoting the absorption and utilization of P, and they play an important role in the P cycle. AKP is responsible for catalyzing the mineralization of organophosphate, which can decompose organophosphate into inorganic P for absorption and use by plants, and this effectively enhances the bioavailability of soil P. A higher pH environment was more conducive to the stability and activity exertion of AKP (Wang et al., 2023c). Previous studies demonstrated that biochar alone or biochar with wood vinegar significantly enhanced soil ALP and AKP because of the higher soil pH (Akmal et al., 2019; Naeem et al., 2021; Zhao et al., 2024). In this study, the application of P-BCL800 decreased ALP activity but improved AKP activity, which may be attributed to the increased pH and AP content in the soil that affected the metabolic environment of microorganisms and further influenced the synthesis and secretion of AKP (Ibrahim et al., 2024). Moreover, biochar, with its large specific surface area and porous structure, stabilized and protected enzymes from degradation or denaturation under different environmental stresses (Elzobair et al., 2016).

In addition, according to correlation coefficients among soil physicochemical properties and chili agronomic traits, P-BCL800 played an important role in enhancing N, P, and K nutrient uptake (Liu et al., 2022b), and enhanced chili growth by balancing the nutrient supply and improving the physicochemical properties of the soil (Bagues et al., 2024; Acharya et al., 2023). Li et al. (2019) (Sharma et al., 2018) demonstrated that Ca^2+^ and Mg^2+^ play important roles in nutrient uptake and plant growth, whereas E_Ca_ and E_Mg_ were capable of regulating soil pH, which is similar to the results of the present study. Moreover, the strong positive correlation between chlorophyll content (SPAD) and Pn indicated that the improved AP in soil could increase SPAD through enhanced Pn (Mahmoud et al., 2022). The application of P-BCL800 markedly improved soil pH and chili growth, inhibited ALP activity, and altered the phosphate mineralization process required for plant growth (Hu et al., 2023). The growth status resulted in an excellent photosynthetic efficiency, which significantly reduced Ci (Guo et al., 2021), thus a negative correlation was observed for ALP and Ci with the other parameters.

### 4.3 P-BCL800 changes soil microbial communities and drives phosphorus cycling

P-BCL800 significantly increased the total and unique OTU numbers. These results are consistent with those of previous studies, which reported that the application of BPF improved soil health by altering soil pH and EC, increasing N, P, and K nutrient availability, enhancing organic matter decomposition, and inhibiting pathogens (El-Naggar et al., 2019; Joseph et al., 2021; Deng et al., 2024). Diverse microbial life was introduced, which enhanced the microbial community in the soil (Zheng et al., 2021; Murtaza et al., 2023).

P-BCL800 application also affected soil microbial alpha and beta diversity. Changes in the Ace and Chao indices in the present study were consistent with the Shannon index, indicating that substituting TPF with P-BCL800 increased species richness, which is in agreement with the results of a previous study (Suleiman et al., 2013). According to Liu et al., the application of biochar or BPF likely enhanced nutrient availability, increased OMC content, altered pH environment, and reduced pathogen activity (Ayaz et al., 2022; Liu et al., 2022b; Zhang and Shen, 2022). The improved soil environment was conducive to the growth of microorganisms (Dai et al., 2021; Wang et al., 2023b). Therefore, the application of BPF enhances soil microbial diversity and uniformity, which may be attributed to the improved availability of N, P, and K nutrients and pH adjustment (Singh et al., 2022). Moreover, species abundance can enhance soil nutrient cycling and promote the absorption of soil nutrients by plants (Yan et al., 2020). There was a significant difference in community composition under the X, Y, and Z treatments, particularly under the Y treatment, which may be attributed to the changes, competition, and interaction of the nutrient supply variations (Sheng et al., 2023). Similar results were observed by Zhao et al (Wang et al., 2022; Zhao et al., 2024), who found that the richness and diversity of the bacterial community markedly increased following the application of biochar.

Soil microbial community changed after P-BCL800 application, and the abundance of Proteobacteria, Actinobacteria, and Alphaproteobacteria increased obviously. X treatment resulted in the highest Alphaproteobacteria abundance (32.38%), and Z treatment resulted in the highest Actinobacteria abundance (13.89%), which may be attributed to the increase in available nutrients for bacterial growth (Fierer et al., 2012). Among them, Proteobacteria and Actinobacteria promote nutrient cycling, and both are capable of solubilizing or adsorbing P in the soil, enhancing the bioavailability of P (Deng et al., 2024; Soumare et al., 2021). Actinobacteria can secrete various enzymes and participate in organic matter degradation and hummus formation (Sun et al., 2014; Mitra et al., 2022). Moreover, Alphaproteobacteria are considered a key microbe for P dissolution and mineralization in soil (Zhao et al., 2024).

Our previous study indicated that the increase in the relative abundance of Patescibacteria and Actinobacteria was directly related to the improvement in soil conditions conducive to the growth and activity of microorganisms (Deng et al., 2024). Microbes, such as Patescibacteria and Actinobacteria, play important roles in the decomposition of organic matter and dissolution of nutrients; thus, their enhancement is important for sustainable agriculture (Singh et al., 2022). According to the results of the RDA between microbial diversity and soil environmental factors, there was a significant positive correlation between AKP activity and pH, and the application of P-BCL800 enhanced AKP activity, likely because an increase in soil pH was conducive to AKP stability and activity, which may decompose organic P into inorganic P, increasing the availability of P (Akmal et al., 2019; Zhao et al., 2024; Wang et al., 2023a).

To summarize, we proposed a synergistic mechanism by which P-BCL800 initially ameliorated the soil physicochemical environment—particularly by increasing pH and organic matter content—which subsequently enriched and stimulated the proliferation of functional microbial taxa with high phosphorus-mobilizing capacity (e.g., Actinobacteria and Alphaproteobacteria). The increased activity of these microbes, as evidenced by elevated AKP activity, accelerated the transformation of recalcitrant phosphorus into plant-available forms (i.e., Olsen-extractable phosphorus, AP), thereby expanding the labile phosphorus pool of soil. This microbiologically driven process established a positive feedback loop that improved phosphorus acquisition, ultimately promoting biomass accumulation and yield improvement. In other words, P-BCL800 indirectly promoted the activities of microorganisms related to the P cycle through influencing soil pH and providing carbon sources, which thereby enhanced P availability. This resulted in a benign ecosystem conducive to the P cycle and chili growth, which is consistent with the results of Liu et al (Liu et al., 2025).

### 4.4 Synthesis and novelty: an integrated microbial-mediated mechanism

The novelty of this study lies in integrating plant growth responses, soil chemistry, and microbial ecology to clarify the mechanism of BPF efficacy in an acidic soil–chili system. We move beyond the established narrative of “biochar improves yield by increasing nutrient retention” to demonstrate that P-BCL800 induces a shift in the soil microbial community toward a more diverse and functionally proficient state, characterized by an increase in the abundance of P-solubilizing taxa (e.g., Actinobacteria and Proteobacteria). This shift is primarily driven by the ability of P-BCL800 to increase soil pH, organic matter, and available P. This microbial-mediated pathway generates a positive feedback loop that sustains nutrient availability, promotes plant health, and improves system resilience.

## 5 Conclusion

In this study, we demonstrated that the application of P-BCL800 had a significant effect on soil physical and chemical properties, microbial community, and chili growth. Under fixed nutrient content, partly replacing the TPF with BPF, a significant improvement was observed in the physical and chemical properties of the soil, including increased soil pH, EC, OMC, AP, and AHN. The abundance of dominant bacterial communities was related to soil environmental factors, and pH, OMC, and AP were the key influencing factors, which promoted the activity of P-solubilizing bacteria to form a benign ecosystem. Consequently, chili growth parameters—including plant height (PH), stem diameter (SD), shoot fresh weight (SFW), root fresh yield (RFY), and fruit quality—were significantly enhanced in three P-BCL800 substitution treatments (33%, 66%, and 100% P-BCL800 replacement) compared to the TPF-only treatment. Among them, the 66% substitution treatment (Y) achieved the highest values across most indicators and exhibited the best overall performance. P-BCL800 not only improved the availability of nutrients, but also regulated the composition and function of the microbial community, thereby promoting the growth of chili and enhancing soil fertility. These components were driven by changes in soil pH, organic matter, and availability of phosphorus. Moreover, the observed increase in soil pH, EC and OMC may highlights the need to balance agronomic benefits with potential long-term risks to soil sustainability. Further long-term field studies are therefore required to systematically evaluate the cumulative effects of P-BCL800 on soil dynamics, salinity development, and crop productivity over time.

## Data Availability

The data presented in the study are deposited in the NCBI repository, accession number:PRJNA1463629. The original contributions presented in the study are included in the article/**Supplementary Material**. Further inquiries can be directed to the corresponding author.
