# Supplementary material for: Evaluation of biochar-based phosphate fertilizer for improving soil properties, chili yield, and microbial function
Source: Front Plant Sci. 2026 May 14;17:1778669. doi: 10.3389/fpls.2026.1778669 (PMC13216040; doi:10.3389/fpls.2026.1778669)

Support Information

Table S1 The physicochemical properties of red soil

|  | pH | EC | Organic matter | alkali-hydrolyzale N | Available P | Rapidly available K | Exchangeable Ca | Exchangeable Mg |
| --- | --- | --- | --- | --- | --- | --- | --- | --- |
| Unit | - | μS/cm | g/kg | mg/kg | mg/kg | mg/kg | mg/kg | mg/kg |
|  | 5.03 | 195 | 4.96 | 29.0 | 1.25 | 20.3 | 81.6 | 20.4 |

**Table S2 The characteristics of biochar**

|  | **Atomic percentage (%)** | | | | | | | | | **Specific surface area (m^2^/g)** | | **pH** | | **EC(mS/cm)** | |
| --- | --- | --- | --- | --- | --- | --- | --- | --- | --- | --- | --- | --- | --- | --- | --- |
|  | C | O | M | P | S | Cl | K | Ca |  | |  | |  | |  |
| BCL800 | 79.25 | 16.60 | 0.72 | 0.22 | 0.27 | 0.25 | 0.81 | 1.88 | 571.43 | | 12.17 | | 12.5 | | |
| P-BCL800 | 75.3 | 21.03 | 0.70 | 1.07 | 0.08 | —— | 0.07 | 1.75 | 517.16 | | 12.09 | | 11.6 | | |

**Table S3 Experimental treatment**

| Treatment | Composition | P-BCL800 (g) | Urea (g) | Potassium dihydrogen phosphate (g) | Potassium sulfate (g) |
| --- | --- | --- | --- | --- | --- |
| CK | No | 0 | 0 | 0 | 0 |
| B | Only P-BCL800 | 17.88 | 0 | 0 | 0 |
| F | Only NaH_2_PO_4_ | 0 | 2.61 | 1.73 | 1.11 |
| X | 33% P-BCL800+67%NaH_2_PO_4_ | 5.96 | 2.61 | 1.15 | 1.48 |
| Y | 66% P-BCL800+34%NaH_2_PO_4_ | 11.92 | 2.61 | 0.58 | 1.85 |
| Z | 100% P-BCL800 | 17.88 | 2.61 | 0 | 2.22 |

Fig. S1 Growth status of chili (a) and fruit morphology (b) after 3 months of planting under different treatments.


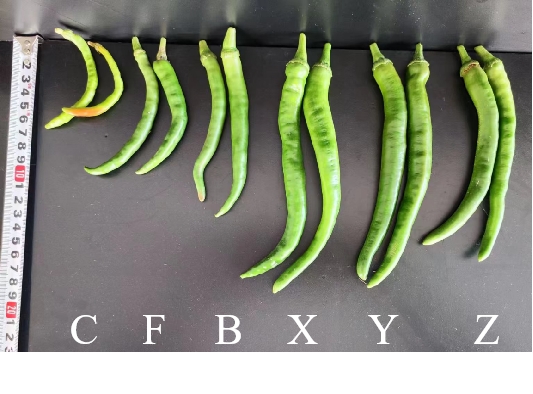

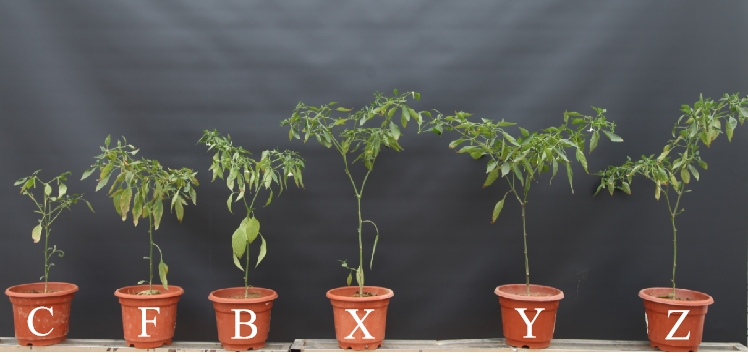


**(a)**

**(b)**

Fig. S2 LDA value


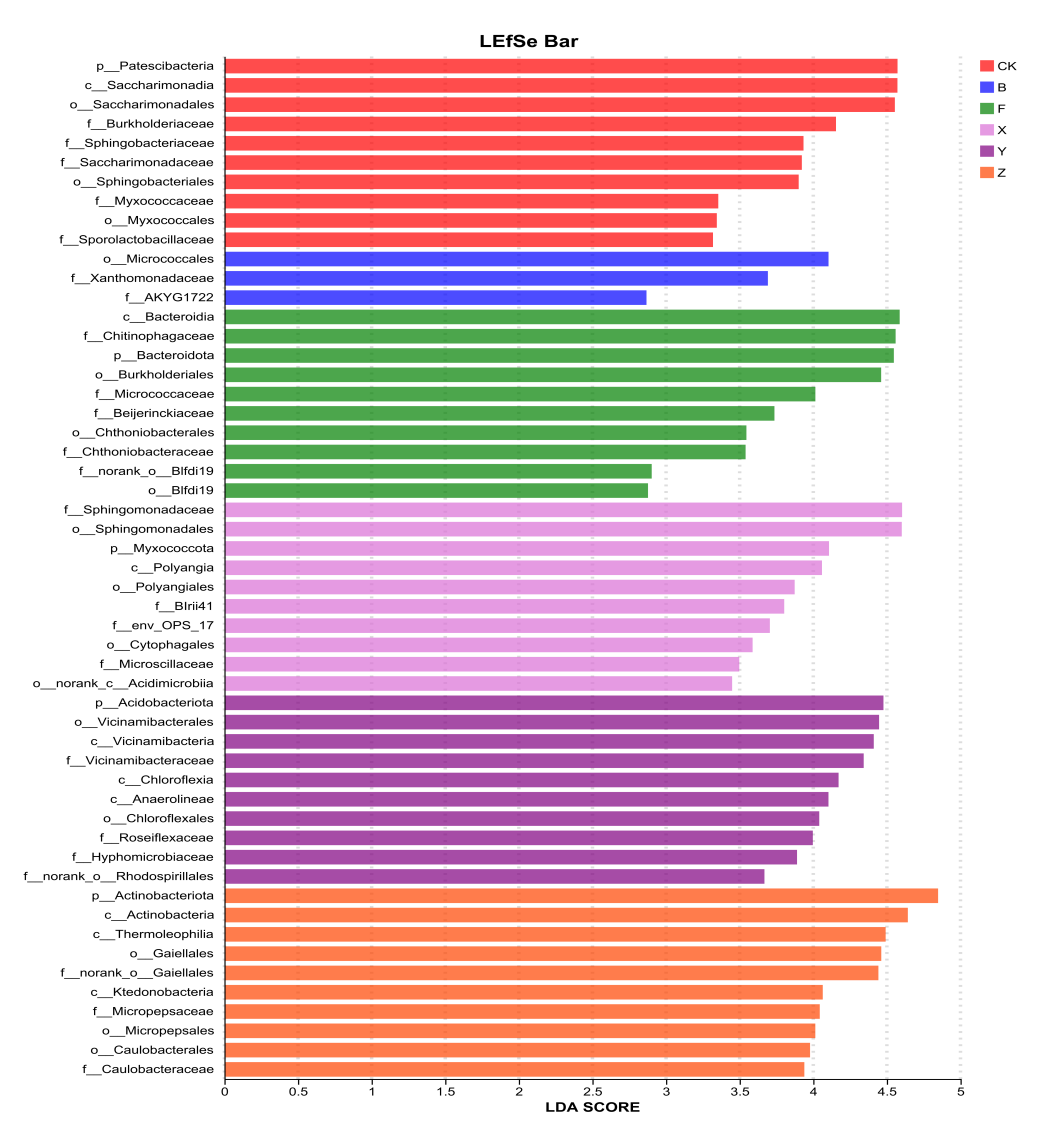

Supplement: Supplementary file 1 [file Supplementaryfile1.doc]
